# A phase 1b dose-escalation/expansion study of BET inhibitor RO6870810 in patients with advanced multiple myeloma

**DOI:** 10.1038/s41408-021-00545-w

**Published:** 2021-09-03

**Authors:** Karthik Ramasamy, Ajay Nooka, Hang Quach, Myo Htut, Rakesh Popat, Michaela Liedtke, Sascha A. Tuchman, Jacob Laubach, Cristina Gasparetto, Asher Chanan-Khan, Mark Hertzberg, Mark deMario, Eveline Nueesch, Evelyne Chesne, Izolda Franjkovic, Katharina Lechner, Martin Kornacker, Hearn Jay Cho

**Affiliations:** 1 Department of Haematology, Oxford University Hospital, NHS Trust, Oxford, United Kingdom; 2 Winship Cancer Institute, Emory University, Atlanta, GA USA; 3 University of Melbourne, St. Vincent’s Hospital, Fitzroy, VIC Australia; 4 City of Hope Comprehensive Cancer Center, Duarte, CA USA; 5 University College London Hospitals, NHS Foundation Trust, London, United Kingdom; 6 Stanford University Cancer Center, Stanford, CA USA; 7 Division of Hematology, Lineberger Comprehensive Cancer Center, University of North Carolina, Chapel Hill, NC USA; 8 Dept. of Medical Oncology, Dana-Farber Cancer Institute, Harvard Medical School, Newton, MA USA; 9 Department of Medicine, Duke Univ. Medical Center, Durham, NC USA; 10 Division of Hematology and Medical Oncology, Mayo Clinic, Jacksonville, FL USA; 11 Department of Clinical Haematology, Prince of Wales Hospital, Sydney, NSW Australia; 12 Roche Pharma Research and Early Development, New York, NY USA; 13 Roche Innovation Center Basel, Roche Pharma Research and Early Development, Basel, Switzerland; 14 Roche Innovation Center Munich, Roche Pharma Research and Early Development, Penzberg, Germany; 15 Tisch Cancer Institute, Icahn School of Medicine at Mount Sinai, New York, NY USA; 16 The Multiple Myeloma Research Foundation, Norwalk, CT USA

**Keywords:** Myeloma, Drug therapy

**Dear Editor**,

The BRD and extra-terminal (BET) family of proteins have been implicated in malignant transformation and in cancer therapy resistance [1]. BET inhibition may reduce transcriptional activation of genes important in the biology of multiple myeloma, including MYC, interferon regulatory factor 4 (IRF4), X-box binding protein 1 (XBP1), and PR domain containing 1 (PRDM1)/B-lymphocyte-induced maturation protein 1 (BLIMP1) [2, 3].

RO6870810 is a novel thienodiazepine, small molecule, non-covalent inhibitor of the BET family of bromodomains. It is optimized from the well-characterized preclinical molecule, JQ1 [4]. RO6870810 potently reduced viability in the majority of the tested cancer cell lines and was shown to inhibit tumor growth in the murine xenograft model KMS-12BM for multiple myeloma. These preclinical datasets provided the necessary rationale for testing RO6870810 in relapsed/refractory myeloma patients, a population for which novel agents and therapeutic approaches remain a significant unmet need.

We report here an international, multicentric phase 1 trial of RO6870810 in patients with advanced multiple myeloma to establish the maximum tolerated dose (MTD)/optimal biological dose (OBD). Twenty-four patients were enrolled in this study, 13 of these were male (54·2%) and 11 female (45·8%). All received at least one dose of RO6870810. Overall, the median age of patients was 65·6 years (range: 46–82 years). Patients had a median of 6 (range 3–9) prior lines of therapy and were refractory to immunomodulatory drugs (IMiDs, 46% of patients), proteasome inhibitors (54%), IMiDs and proteasome inhibitors (38%), and daratumumab (33%) (Supplementary Table 1).

In the dose-escalation part of the study, 13 patients were treated with RO6870810 across ascending dose levels (0·30, 0·45, and 0·65 mg/kg). Overall, 31 treatment cycles were administered in Part 1 of the study (Supplementary Table 2). In the dose-expansion part of the study (Part 2), 11 patients were treated at a dose of 0·65 mg/kg.

The 24 enrolled patients experienced a total of 319 AEs. All patients in the study experienced at least one AE that was considered related to the study treatment by the Investigator. A majority of patients experienced at least one grade ≥3 AE (21 patients [87·5%]) and/or serious adverse event (SAE) (13 patients [54·2%]). Three patients (12·5%) experienced a total of four AEs leading to discontinuation of the study treatment (left ventricular dysfunction, fatigue, sepsis, and staphylococcal bacteremia; the latter two AEs occurred in the same patient).

A total of eight deaths (33·3%) were reported in the study, all as a consequence of progressive disease. One of these was reported as a fatal AE of renal impairment secondary to disease progression (Supplementary Table 3).

DLTs were not observed at the first two dose levels. One patient in the 0·65 mg/kg cohort in Part 1 experienced a related grade 3 AE of angina pectoris and grade 4 AE of thrombocytopenia, both of which qualified as DLT (Supplementary Tables 2 and 4). The event of thrombocytopenia led to treatment interruption. At the last reporting, the event of angina pectoris was resolving with sequelae and the event of thrombocytopenia had resolved. An angiogram was not performed. 0·65 mg/kg was the maximum planned dose and since MTD was not reached, 0·65 mg/kg was utilized as the dose for the expansion cohort.

The most common treatment-emergent AEs, experienced by ≥30% patients, were injection site reaction (19 patients [79·2%]), fatigue (15 patients [62·5%]), anemia (12 patients [50·0%]), thrombocytopenia (11 patients [45·8%]), nausea (ten patients [41·7%]), diarrhea (nine patients [37·5%]), and decreased appetite (eight patients [33·3%]). (Supplementary Table 5).

Grade ≥3 AEs occurred in 21 (87·5%) patients (59 events in total). The AEs experienced by ≥5% of patients were thrombocytopenia (nine patients [37·5%]), anemia (seven patients [29·2%]), fatigue (three patients [12·5%]), injection site reaction, malaise, decreased appetite, hyponatremia, and platelet count decreased (two patients [8·3%] in each preferred term [PT]).

Approximately half (13 patients [54·2%]) of the enrolled patients experienced a total of 31 SAEs. The SAEs experienced by ≥5% of patients were thrombocytopenia (three patients [12·5%]), injection site reaction, acute kidney injury, and malaise (two patients [8·3%] in each PT). One case of acute kidney injury was assessed as treatment-related. Seven patients (29·2%) experienced 12 treatment-related SAEs. The related SAEs experienced by >1 patient were injection site reaction and malaise (two patients [8·3%] in each PT).

Chromatin immunoprecipitation sequencing previously identified a strong BRD4-driven super-enhancer near the CD11b promoter, and displacement of bound BRD4 from the super-enhancer element by a BET inhibitor (BETi) results in diminished CD11b gene expression. In this study, CD11b expression in monocytes was measured by flow cytometry pre-dose and at various time points in cycle 1. Treatment with RO6870810 led to sustained decreases in CD11b during the dosing period across all dose levels (Fig. 1).

**Fig. 1:**
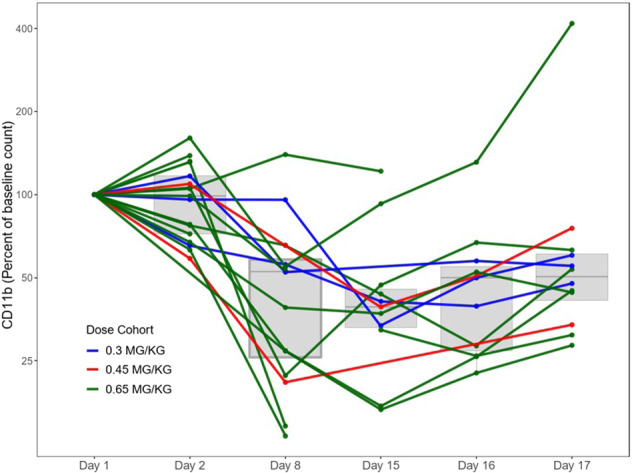
Flow cytometry analysis of percent change in CD11b expressing monocytes from baseline count. In cases of delayed sampling, the actual time points are shown.

The ORR was 16·7%; of the 24 patients treated in the study, four patients achieved a PR, one of which was reported 12 weeks after the end of study treatment (Supplementary Table 6). No complete responses or very good partial responses were achieved. One patient experienced a minimal response. The clinical benefit rate (minimal response or better) was 20·8% (five patients). 17 patients (70·8%) achieved stable disease or better. DoR ranged between 5·3 and 6.0 weeks. There was no evidence for a dose-related increase in efficacy, but numbers are too low to draw conclusions.

All 24 enrolled patients discontinued the study treatment. The primary reasons for discontinuation of the study treatment were progressive disease in 12 patients (50.0%) and withdrawal by subject in eight patients (33·3%).

Several BET inhibitors (BETis) are currently under investigation for the treatment of both solid tumors and hematological malignancies, and varying degrees of activity and tolerability have been reported [5–7]. In this first-in-human trial of the BETi RO6870810, we have established 0·65 mg/kg as the recommended monotherapy dose in relapsed/refractory multiple myeloma. The safety profile of RO6870810 was comparable with safety data reported for other BETi [5] with a predominance of hematological and gastrointestinal toxicities. A high incidence of grade 3–4 thrombocytopenia and grade 3 anemia was observed. However, none of these events led to study drug discontinuation. While cytopenias are a known side effect of BETis, advanced myeloma and extensive prior therapy likely impaired hematopoietic reserve and increased sensitivity to myelosuppression and resultant cytopenias. In a similar trial with RO6870810 for patients with acute myeloid leukemia, the MTD was determined to be 0·45 mg/kg in a 21-day cycle, based on DLTs observed at 0·65 mg/kg (hypertension, anorexia, and fatigue) [8]. In that trial, cytopenias were rarely noted as AEs, and only febrile neutropenia (37·5% of patients) occurred in more than 10% of patients.

Nausea, diarrhea, and decreased appetite occurred at high frequency and were not prevented by the SC administration of RO6870810, suggesting that these gastrointestinal AEs are not impacted by route of administration.

Decreases in CD11b in peripheral blood mononuclear cells were observed with RO6870810 treatment, in line with the putative mechanism of action of RO6870810 preventing BET co-activator loading at super-enhancers. Reductions in CD11b were most pronounced during the end of the 14-day dosing period in cycle 1 but were similar across dose groups and showed limited interpatient variability. Thus, the use of CD11b expression as a pharmacodynamic marker to guide dosing and to predict response may be limited.

While our pharmacodynamic findings provided evidence for on-target effects of RO6870810, clinical response rates with RO6870810 were infrequent and of short duration when they occurred. This is in line with the preliminary activity of RO687081 observed in the first-in-human dose-escalation trial where ORRs were 25% (2/8), 2% (1/47), and 11% (2/19) for patients with nuclear protein of the testis carcinoma (NUT carcinoma), other solid tumors, and diffuse large B-cell lymphoma (DLBCL), respectively [9]. Limited clinical data exist on the use of other BET inhibitors in multiple myeloma. A phase 1 trial with OTX015 resulted in a few responses in patients with DLBCL while the best response achieved in the 12 patients with myeloma was stable disease (17%) or progressive disease (83%) [10].

While treatment with RO6870810 was discontinued due to AEs only in three patients, it could be argued that the overall limited tolerability of RO6870810 at 0·65 mg/kg, with eight patients withdrawing from study treatment, may have resulted in insufficient exposure to study drug to achieve a higher response rate. Further examination of the lower dose levels in a more robust patient population may yield longer treatment periods and improve clinical outcomes.

There is currently limited evidence that BET inhibition alone results in relevant clinical activity against multiple myeloma. Studies show the synergistic activity of histone deacetylase inhibitors and BETis in preclinical setting [11, 12]. One approach to explore is, combining different therapies that act on epigenome, such as panobinostat, which is an approved therapeutic agent for relapsed myeloma in both the United States and the European Union. An alternative approach is to exploit the immunoregulatory function of BETis by combining them with IMiDs and/or anti-CD38 antibody therapies [13]. Careful titration of BETis in rational Phase 1b combination trials supported by preclinical synergy can be considered to optimize the risk/benefit profile.

## Data Sharing

Qualified researchers may request access to individual patient-level data through the clinical study data request platform (https://vivli.org/). Further details on Roche’s criteria for eligible studies are available here (https://vivli.org/members/ourmembers/). For further details on Roche’s Global Policy on the Sharing of Clinical Information and how to request access to related clinical study documents, see here (https://www.roche.com/research_and_development/who_we_are_how_we_work/clinical_trials/our_commitment_to_data_sharing.htm).

## Supplementary information

Supplementary Information
