# Supplementary material for: A phase 1b dose-escalation/expansion study of BET inhibitor RO6870810 in patients with advanced multiple myeloma
Source: Blood Cancer J. 2021 Sep 3;11(9):149. doi: 10.1038/s41408-021-00545-w (PMC8417099; doi:10.1038/s41408-021-00545-w)
Supplement: Supplementary file 1 — Supplementary Information [file 41408_2021_545_MOESM1_ESM.pdf]

Supplement

Supplementary table 1: Baseline patient characteristics

Parameter	
Eligible patients (study population), n	24
Age, median, years	65·5 (range: 46 - 82)
Male/female, n (%)	13 (54·2) / 11 (45·8)
ECOG PS, n (%)<br>0<br>1<br>2	 10 (41·7)<br>13 (54·2)<br>1 (4·2)
Prior lines of therapy,<br>median (min-max)	6 (3-9)
Refractory to<br>Immunomodulatory drugs<br>Proteasome inhibitors<br>Immunomodulatory drugs and proteasome inhibitors<br>Daratumumab	 46%<br>54%<br>38%<br>33%

ECOG PS = Eastern Cooperative Oncology Group Performance Status.

Supplementary table 2: Summary of dose-escalation

Cohort	Dose	Patients	Total Cycles Completed, n	DLT, n of pts
**1**	0·30 mg/kg	3	7	0
**2**	0·45 mg/kg	3	6	0
**3**	0·65 mg/kg	7	18	1 (Grade 4 thrombocytopaenia, grade 3 angina pectoris)
**Total**		**13**	**31**	**1**

***Supplementary table* 3: Safety summary**

	**Patients With ≥1 AE, n (%)**
**Any AE**	**24 (100·0)**
Grade ≥3 AE	21 (87·5)
Grade 5 AE	1 (4·2)
**Treatment-related AE**	**24 (100·0)**
Grade ≥3 TRAE	17 (70·8)
Grade 5 TRAE	0
**Any SAE**	**13 (54·2)**
Treatment-related SAE	7 (29·2)
**AE leading to treatment discontinuation**	**3 (12·5)**
TRAE leading to treatment discontinuation	2 (8·3)

Supplementary Table 4: DLT criteria

SOC Disorder	CTCAE Term	DLT Criteria
Blood and lymphatic system disorders	Neutropenia	Grade 4 neutropenia lasting ≥ 5 consecutive days.
	Febrile neutropenia	Grade ≥ 3 neutropenia associated with single body temperature > 38.3°C or sustained body temperature ≥ 38°C for > 1 hour, and/or with infection.
	Thrombocytopaenia	Grade 4 thrombocytopaenia or grade 3 thrombocytopaenia associated with bleeding episodes.
	Anaemia	Grade ≥3 anaemia with hemolysis.
General disorders and administration site conditions	Injection site reaction	Grade 3 or 4 skin ulceration or 'other' skin and subcutaneous tissue disorders related to the subcutaneous injection of RO6870810.
Other non-hematologic disorders	Other adverse events	Grade ≥ 3 non-hematological toxicity, excluding:<br>Fatigue (i.e., asthenia) and malaise that resolve to grade ≤ 2 within 1 week.<br>An isolated, asymptomatic elevation of unconjugated bilirubin with normal other liver function tests (LFTs) and in the absence of hemolysis.<br>Grade 3 nausea, vomiting or diarrhoea including their clinical sequelae (e.g., fluid loss with subsequent dehydration, electrolyte loss (sodium, potassium, magnesium, chloride) occurring with sub-optimal prophylactic and curative treatment and that are responding to supportive care within 72 hours).<br>A drug-related grade ≤ 3 fever.<br>Grade 3 neuropathy if the patient began therapy with a grade 2 neuropathy at baseline.<br>Alopecia.
Other DLT criteria:	Delay of the next scheduled administration of > 2 weeks (monotherapy) or 4 weeks (combination) due to either treatment-related AE or clinically significant laboratory abnormality.<br>Any other treatment-related toxicity considered significant enough to be qualified as a DLT in the opinion of the Investigators after discussion with the Sponsor	

***Supplementary table 5*: Most common (≥ 30%) treatment-emergent AEs (N = 24)**

	**Any-Grade, n (%)**	**Grade ≥ 3, n (%)**
**Injection site reaction**	19 (79·2)	2 (8·3)
**Fatigue**	15 (62·5)	3 (12·5)
**Anaemia**	12 (50·0)	7 (29·2)
**Thrombocytopaenia**	11 (45·8)	9 (37·5)
**Nausea**	10 (41·7)	-
**Diarrhoea**	9 (37·5)	-
**Decreased appetite**	8 (33·3)	2 (8·3)

Supplementary table 6: **Response rates**

**Parameter**	**Category**	**Part 1**			**Part 2**
		**0.3 mg/kg**	**0.45 mg/kg**	**0.65 mg/kg**	**0.65 mg/kg**
**Overall Best Response**	Number of patients	3	3	7	11
	PR	1* (33%)	0 (0%)	2 (28·6%)	1 (9·1%)
	MR	0 (0%)	0 (0%)	0 (0%)	1 (9·1%)
	SD	1 (33%)	3 (100%)	3 (42·9%)	5 (45·5%)
	PD	1 (33%)	0 (0%)	0 (0%)	3 (27·3%)
	Missing	0 (0%)	0 (0%)	2 (28·6%)	1 (9·1%)
	ORR (CR + VGPR + PR)	1 (33%)	0 (0%)	2 (28·6%)	1 (9·1%)
	CBR (CR + VGPR + PR + MR	1 (33%)	0 (0%)	2 (28·6%)	2 (18·2%)
	DCR (CR + VGPR + PR + MR + SD)	2 (66%)	3 (100%)	5 (71·4%)	7 (63·6%)

CBR = clinical benefit rate; CR = complete response; DCR = disease control rate; MR = minimal response; ORR = overall response rate; PD = progressive disease; PR = partial response; SD = stable disease; VGPR = very good partial response.

*This partial response was reported 12 weeks after end of study treatment.

Supplementary Information – Material and Methods

Study Design and Participants

This was a phase 1b, open-label, multicenter, global study designed to assess the safety and tolerability of RO6870810 as monotherapy in patients with relapsed/refractory multiple myeloma.

Patients eligible for study inclusion included patients who had symptomatic multiple myeloma as defined by the International Myeloma Working Group (IMWG)[20] who had either progressed on at least three prior lines of therapy including a proteasome inhibitor and an immunomodulatory agent, or was double-refractory to a proteasome inhibitor and an immunomodulatory agent. Patients had measurable disease defined as serum M-protein ≥1 g/dL and/or urine M-protein ≥200 mg/24h and/or serum free-light chain (SFLC) assay ≥ 10 mg/dL (≥100 mg/L) and an abnormal SFLC ratio (<0·26 or >1·65). Key exclusion criteria were plasma cell leukemia, POEMS syndrome, known amyloidosis and, for the expansion part only, primary refractory myeloma. All patients were 18 years of age or older, had an Eastern Cooperative Oncology Group performance status ≤2, and had acceptable organ function with a life expectancy of at least 3 months. Further details are provided in the protocol.

This study was approved by local institutional review boards and conducted in accordance with the protocol, Good Clinical Practice standards, and the Declaration of Helsinki. All enrolled patients gave written informed consent. The trial was registered with ClinicalTrials.gov, NCT03068351.

Procedures

The study was conducted in 2 parts. Part 1 consisted of a dose-escalation to evaluate the safety, tolerability and Dose Limiting Toxicities (DLTs), and to establish the maximum tolerated dose (MTD)/optimal biological dose (OBD) of RO6870810 as monotherapy. In

Part 2, the safety, tolerability and activity of RO6870810 were further evaluated in an expansion patient cohort, at the dose defined in Part 1.

In Part 1, patients received subcutaneous (SC) escalating doses of RO6870810 (0·30, 0·45, and 0·65 mg/kg) in a standard 3 + 3 design on Days 1-14 of 21-day cycles.

In Part 2, patients in the expansion cohort received RO6870810 as monotherapy at the dose determined in Part 1. Patients continued therapy until the occurrence of disease progression or unacceptable toxicity.

The MTD was defined as the dose-level at which a DLT was seen in 2 or more out of 6 patients, minus one dose-level. A DLT was defined as a grade ≥3 non-hematological or hematological toxicity that occurred during the predefined DLT assessment period of 21 days from the first administration of RO6870810, and was considered by the Investigator to be related to study treatment. Patients who failed to start the second cycle of therapy within 2 weeks of end of the first cycle due to either treatment-related adverse events (AE) or laboratory abnormality were also counted as DLT events. Complete DLT criteria can be found in supplementary table 6.

Outcomes

The primary objectives in the dose-escalation phase were to determine the safety, tolerability, and MTD/OBD of RO6870810 monotherapy. For the dose expansion phase, the primary objective was to preliminarily evaluate the clinical activity of RO6870810 given as monotherapy by estimating objective response rate (ORR), progression-free survival (PFS), duration of response (DoR) and overall survival (OS) utilizing the IMWG uniform response criteria.

The secondary objectives of this study were 1) to evaluate preliminary evidence of antitumor activity in the dose-escalation phase, before broader testing in the dose expansion phase, 2) to further characterize the single- and multiple-dose pharmacokinetics (PK) of RO6870810 and its potential metabolites when given as monotherapy, and 3) to further characterize the overall safety profile of RO6870810 monotherapy in the dose expansion phase.

Patients were considered evaluable for safety if they received ≥1 dose of study drug. All AEs were graded per the National Cancer Institute's Common Terminology Criteria for Adverse Events version 4.03.

The safety measurements for this study included nature and frequency of DLTs and DLT-equivalent toxicities, incidence, nature and severity of AEs, and changes in vital signs, physical findings, ECG parameters, and clinical laboratory results during and following RO6870810 administration.

Disease-specific outcome measures were assessed by ORR, defined as the proportion of patients who achieved confirmed partial response (PR) or better as defined by IMWG uniform response criteria (Kumar S, Paiva B, Anderson KC, et al. International Myeloma Working Group consensus criteria for response and minimal residual disease assessment in multiple myeloma. Lancet Oncol 2016; 17: e328−46). Biochemical response assessments were completed at end of every cycle and included measurements of M-protein and light chains.

Patients who received ≥1 dose of study drug were considered evaluable for efficacy. Patients with missing or non-evaluable response assessments were classified as non-responders.

Among patients achieving response, DoR was defined as the time from the first observed PR or better, until the time of disease progression or death. If a patient did not experience death or disease progression before the end of the study, DoR was censored at the day of the last disease assessment. If no assessments were performed after first documented response, the patient was censored at the date of the first occurrence of the response plus one day.

PFS was defined as the time from the first day of study treatment to disease progression or death, whichever occurred first. If a patient had not experienced disease progression or death, PFS was censored on the day of the last tumor assessment. Patients with no post-baseline tumor assessments were censored at the date of first study treatment plus one day.

OS was defined as the time from the first day of study treatment to death from any cause. Data for patients who had not died were censored at the date the patient was last known to be alive.

For the pharmacodynamic assessments, venous blood samples were collected at cycle 1 Days 1, 2, 8, and 15. CD11b expression in monocytes was assessed as a surrogate pharmacodynamic marker using flow cytometry before and after the first administration of RO6870810.

Statistical analysis

Formal hypothesis testing was not performed for this phase 1b study. The sample size was based on a standard 3 + 3 dose-escalation design and was considered sufficient to evaluate the safety and clinical activity of RO6870810. AEs were coded using the current Medical Dictionary for Regulatory Activities, and safety and preliminary clinical efficacy were summarized using descriptive statistics. The 95% confidence interval for the ORR was calculated using the exact Clopper-Pearson method with continuity correction, and PFS was analyzed using the Kaplan-Meier method. Percentage changes in CD11b from baseline by flow cytometry were summarized with descriptive statistics and plotted over time.

Role of the funding source

TBC: The sponsor of the study (F. Hoffmann La Roche Ltd) supplied RO6870810, contributed to the study design, was involved in collection and analysis of data, and writing of the report. All authors had full access to all the data upon request and were involved in data interpretation, preparation of the letter, revision, and final approval. The corresponding author had the final responsibility to submit for publication.
